# Key pathways and genes controlling the development and progression of clear cell renal cell carcinoma (ccRCC) based on gene set enrichment analysis

**DOI:** 10.1007/s11255-013-0511-2

**Published:** 2013-08-14

**Authors:** Haipeng Huang, Yanyan Tang, Wenwu He, Qi Huang, Jianing Zhong, Zhanbin Yang

**Affiliations:** 1 Department of Urinary Surgery, The First Affiliated Hospital, Guangxi Medical University, 6th Shuangyong Road, Nanning, 530021 Guangxi China; 2 Department of Neurology, First Affiliated Hospital, Guangxi Medical University, Nanning, Guangxi China; 3 Department of Cardiothoracic Surgery, Nanchong Central Hospital, The Second Clinical College of North Sichuan Medical College, Nanchong, Sichuan China; 4 Guilin Medical College, Guilin, Guangxi China

**Keywords:** Clear-cell renal cell carcinoma (ccRCC), Gene set enrichment analysis, Meta-analysis, Key pathways

## Abstract

**Background:**

Clear-cell renal cell carcinoma (ccRCC) is one of the most common types of kidney cancer in adults; however, its causes are not completely understood. The study was designed to filter the key pathways and genes associated with the occurrence or development of ccRCC, acquaint its pathogenesis at gene and pathway level, to provide more theory evidence and targeted therapy for ccRCC.

**Methods:**

Gene set enrichment analysis (GSEA) and meta-analysis (Meta) were used to screen the critical pathways and genes which may affect the occurrence and progression of ccRCC on the transcription level. Corresponding pathways of significant genes were obtained with the online website DAVID (http://david.abcc.ncifcrf.gov/).

**Results:**

Thirty seven consistent pathways and key genes in these pathways related to ccRCC were obtained with combined GSEA and meta-analysis. These pathways were mainly involved in metabolism, organismal systems, cellular processes and environmental information processing.

**Conclusion:**

The gene pathways that we identified could provide insight concerning the development of ccRCC. Further studies are needed to determine the biological function for the positive genes.

## Introduction

Renal cell carcinoma (RCC) is one of the most common genitourinary malignancies, accounting for about 3 % of all cancers worldwide [1]. Clear cell renal cell carcinoma (ccRCC) is the most common histological type of renal cell carcinoma, also called conventional RCC, which represents 75–80 % of RCC. The male/female ratio is approximately 2:1 [2]. Initial treatment is most commonly a radical or partial nephrectomy and remains the mainstay of curative treatment [3]. Where the tumor is confined to the renal parenchyma, the 5-year survival rate is 60–70 %, but this is lowered considerably where metastases have spread. It is relatively resistant to radiation therapy and chemotherapy, although some cases respond to immunotherapy.

After the completion of the Human Genome Project, advances in microarray technology led to global gene expression profiling of ccRCC [4–6]. Microarray technology profiles the expression levels of thousands of genes simultaneously, providing a snapshot of transcript levels in the cells/tissues being studied; it is a powerful tool to study ccRCC. All microarray data are available from the Gene Expression Omnibus (GEO) public database at NCBI [7]. However, the large amounts of data acquired must be reduced or ‘translated’ to a smaller set of genes representing meaningful biological differences between control and test systems and validated in an experimental or clinical setting. It is a challenge to analyze such high information from microarray datasets to identify molecular pathways and key genes deregulated in ccRCC. To resolve this conflict, Subramanian describes a method gene set enrichment analysis (GSEA) which has been recognized as a breakthrough for the gene set and functional pathways analysis [8]. GSEA is a method that allows us to search the key genes and pathways associated with the occurrence and development of disease by analyzing diverse experimental datasets. However, the difference of platforms, the sample capacity and the standardization affect the result more or less. Meta-analysis of microarray data can be a better way to solve the problem of poor reproducibility and reliability [9]. In our study, after a standardized microarray preprocessing for all the expression datasets, GSEA and a meta-analysis were used to detect the mixing pathways and key genes which can provide the theoretical basis to the further perception of the biological mechanism of ccRCC.

## Materials and methods

### Data collection

We used clear cell renal cell carcinoma as the keywords and set the limit of study type expression profiling by array and a species limit of humans to search GEO (http://www.ncbi.nlm.nih.gov/geo/) for the relevant gene datasets. Search results provided 2,762 identified datasets involved in ccRCC. Studies that met all of the following criteria were included: (1) the data were about genome-wide RNA expression; (2) the complete microarray raw or normalized data were effective; (3) the data provided a comparison of renal tissue between ccRCC patients and normal controls relatively; (4) datasets contained more than three samples; (5) the raw data were expressed as CEL files. Finally, there were three gene expression datasets which met the selection criteria (Table 1).

**Table 1:** Characteristics of datasets selected in the studies

GEO accession	Contributor	Years	Chip	Experimental design	Probs	Source	Disease	Normal
GSE781	Lenburg ME	2003	HG-U133A	Paired, tissues	22,283	Homo sapiens	7	7
GSE6344	Gumz ML	2007	HG-U133A	Paired, tissues	22,283	Homo sapiens	10	10
GSE36895	Peña-Llopis S	2012	HG-U133_Plus_2	Paired, tissues	54,675	Homo sapiens	23	23

Paired = compare clear cell renal carcinomas to normal controls from the same patients with clear cell renal carcinoma

### Gene set enrichment analysis

The category version 2.10.1 package was used to perform with GSEA. General statistical analysis and computing was processed in the R statistical programming language [10]. The Robust Multichip Averaging (RMA) [11] algorithm in the affy conductor package [12] was used for each affymetrix raw dataset to calculate background-adjusted, normalized and log2 probe set intensities. The only genes we selected should have been mapped to an explicit KEGG pathway for the purpose of analyzing the GSEA and meta-analysis further [13]. We performed pathway analysis of each dataset independently. The measure of variability was within the interquartile range (IQR) and a cut-off was set up to remove IQR values under 0.5 for all the remaining genes. If one gene was targeted for multiple probe sets, we retained the probe set with the largest variability. Genes in each pathway went through the Student’s *t* test, and each pathway’s *p* value was obtained in the permutation test with 1,000 times. The *p* value was not more than 0.05.

### Meta-analysis

To obtain the differentially expressed genes from the remaining genes of each dataset above, meta-analysis was carried out in SAS 9.13. The following formula was applied to calculate chi-square value of each gene [14]:

$$ X^{2} = - 2\sum\limits_{i = 1}^{\text{k}} {\log_{\text{e}} P^{\text{i}} } $$

(K is the number of the datasets). 

To calculate *p* value of each gene, and retained the genes with *p* < 0.05. Significant genes were used to obtain the pathways of the KEGG from DAVID Bioinformatics Resources 6.7 (http://david.abcc.ncifcrf.gov/).

## Results

### GSEA analysis

According to the inclusion criteria, we obtained three datasets in the end. Tissues used to extract the total RNA were matched pairs from clear cell renal cell carcinoma and normal tissue adjacent to renal cell carcinoma. The fuhrman grade of ccRCC was no more than three. Due to the samples used, genomic profiling were matched pairs. It reduced the influence of multiple factors on GSEA and Meta-analysis, ensuring the reliability of the obtained conclusion.

The three inclusion datasets contained 40 ccRCC cases and 40 controls. GSEA method was used separately on each dataset to find the significantly changed genes and the significant co-pathways. After GSEA analysis, 8,506 significantly changed genes were screened out from the three gene expression microarray datasets. Overlap existed in the up-regulated and down-regulated pathways. There were fourteen mixing pathways including 206 up-regulated and 253 down-regulated pathways from three datasets. Detailed information about the analysis results is shown in Table 2.

**Table 2:** Summary of each dataset used gene set enrichment analysis (GSEA)

Studies	Number of patients	Number of controls	Number of significantly changed genes after GSEA	Up-regulated pathways	Down-regulated pathways
GSE781	7	7	2,689	73	57
GSE6344	10	10	2,644	68	96
GSE36895	23	23	3,173	65	100

### Meta-analysis

To further identify the results above, meta-analysis was used to detect differentially expressed genes between two experimental groups. We got the *p* value for each gene based on unpaired *t* test. A total of 1,150 significant genes were detected (*p* < 0.05). Furthermore, the Database for Annotation, Visualization and Integrated Discovery (DAVID) (http://david.abcc.ncifcrf.gov/) was utilized for the annotation of these genes. We imported the official gene symbols of 1,150 genes into the gene functional classification tool of DAVID. In order to identify biologically relevant molecular networks of these genes, KEGG (http://www.genome.jp/kegg/), a distinct pathway analysis tools of bioinformatics endowed with comprehensive knowledgebase was used. There were 1,038 genes identified by KEGG. In total, 48 KEGG pathways were detected. More details were shown in Table 3.

**Table 3:** Common significant pathways were obtained from 3 clear cell renal carcinoma tissue datasets by meta-analysis

Pathway entry	Pathway names	*p* value	Number of genes expressed in the pathways	Up- or down-regulated pathways
map00280	Valine, leucine and isoleucine degradation	8.79E−14	33	Up
map00020	Citrate cycle (TCA cycle)	1.55E−10	24	Up
map00010	Glycolysis/gluconeogenesis	1.41E−08	33	Up
map00640	Propanoate metabolism	3.35E−08	22	Up
map00071	Fatty acid metabolism	2.58E−07	24	Up
map00620	Pyruvate metabolism	1.34E−06	23	Up
map00650	Butanoate metabolism	5.21E−06	20	Up
map00330	Arginine and proline metabolism	1.16E−04	24	Up
map00190	Oxidative phosphorylation	4.13E−04	44	Up
map00260	Glycine, serine and threonine metabolism	4.36E−04	16	Up
map05012	Parkinson’s disease	5.80E−04	43	Up
map04514	Cell adhesion molecules (CAMs)	5.95E−04	44	Down
map03320	PPAR signaling pathway	6.52E−04	27	Up
map05416	Viral myocarditis	0.00107	27	Down
map00380	Tryptophan metabolism	0.00119	18	Up
map04142	Lysosome	0.00129	39	Up
map00410	beta-Alanine metabolism	0.00183	12	Up
map00100	Steroid biosynthesis	0.00292	10	Up
map04610	Complement and coagulation cascades	0.00363	25	Up
map00051	Fructose and mannose metabolism	0.00451	15	Down
map04612	Antigen processing and presentation	0.00605	28	Down
map05219	Bladder cancer	0.00611	17	Down
map05212	Pancreatic cancer	0.00669	25	Down
map00670	One carbon pool by folate	0.00782	9	Up
map05330	Allograft rejection	0.00819	15	Down
map00310	Lysine degradation	0.01017	17	Up
map05110	Vibrio cholerae infection	0.01223	20	Up
map00903	Limonene and pinene degradation	0.01281	8	Up
map05130	Pathogenic *Escherichia coli* infection	0.01493	20	Down
map04940	Type I diabetes mellitus	0.01510	16	Down
map05010	Alzheimer’s disease	0.01525	46	Up
map04540	Gap junction	0.01631	28	Down
map00630	Glyoxylate and dicarboxylate metabolism	0.01987	8	Up
map00072	Synthesis and degradation of ketone bodies	0.02115	6	Down
map05214	Glioma	0.02205	21	Down
map05222	Small cell lung cancer	0.02571	26	Down
map04512	ECM–receptor interaction	0.02571	26	Down
map05120	Epithelial cell signaling in helicobacter pylori infection	0.02621	22	Up
map04510	Focal adhesion	0.03278	53	Down
map00250	Alanine, aspartate and glutamate metabolism	0.03679	12	Up
map04666	Fc gamma R-mediated phagocytosis	0.03719	28	Down
map04360	Axon guidance	0.03811	36	Down
map04012	ErbB signaling pathway	0.03873	26	Up
map05332	Graft-versus-host disease	0.04031	14	Down
map05200	Pathways in cancer	0.06820	79	Down
map00270	Cysteine and methionine metabolism	0.06916	12	Up
map05320	Autoimmune thyroid disease	0.08014	16	Down
map05322	Systemic lupus erythematosus	0.09307	27	Down

### The results of GSEA and meta-analysis

To search the intersection pathways, a comparative analysis was made subsequently between the significant common pathways of GSEA and meta-analysis. At last, 37 consistent pathways and significant genes (*p* < 0.05) in these pathways were obtained. These pathways mainly concerned metabolism, organismal systems, cellular processes, environmental information processing, and human diseases. The details are shown in Table 4.

**Table 4:** Common crossing significant pathways and genes between the results of GSEA and meta-analysis

Pathway entry	Pathway names	Classification	Up- or down-regulated	*p* value	Number of key genes	Included genes (*p* value)
map00020	Citrate cycle (TCA cycle)	Metabolism; carbohydrate metabolism	up	1.55E−10	24	DLST(1.58E−05), ACO2(0.009461), SUCLG2(0.001127), ACO1(3.37E−08), SUCLG1(7.68E−13), OGDHL(7.95E−10), ACLY(6.53E−05), OGDH(0.015859), PCK2(3.66E−08), PDHB(3.11E−07), PCK1(8.90E−06), SDHB(0.000273), IDH3G(0.000767), SDHC(0.002922), SDHD(0.002733), DLD(0.001570), IDH2(4.67E−07), IDH1(4.72E−05), PDHA1(3.64E−07), SUCLA2(0.002727), MDH2(0.041430), MDH1(3.71E−05), FH(8.33E−05), PC(2.46E−08)
map00071	Fatty acid metabolism	Metabolism; lipid metabolism	up	2.58E−07	24	ACAA2(0.015364), ACOX1(1.77E−07), GCDH(2.55E−07), ACADSB(1.06E−05), ACADM(0.000825), CPT2(0.012060), ACADS(0.000351), EHHADH(0.009979), ADH6(6.28E−10), ECHS1(1.41E−09), ADH1B(1.11E−05), ACAT1(3.35E−05), PECI(0.000010), ALDH3A2(0.001311), CPT1A(0.028547), CYP4A11(3.26E−05), ALDH7A1(0.000678), CYP4A22(5.91E−05), ALDH1B1(1.21E−06), ALDH2(2.04E−05), HADH(0.009979), ALDH9A1(7.68E−06), ACAA1(6.20E−09), ACSL5(0.000549)
map00100	Steroid biosynthesis	Metabolism; lipid metabolism	up	0.00292	10	TM7SF2(8.34E−08), CEL(0.000000), EBP(0.000483), CYP27B1(1.05E−07), LIPA(1.42E−08), CYP51A1(0.006793), DHCR7(0.038355), HSD17B7(0.017025), SC5DL(2.60E−05), FDFT1(0.000663)
map00190	O idative phosphorylation	Metabolism; energy metabolism	Up	4.13E−04	44	UQCRC1(1.72E−10), NDUFB8(0.026633), ATP5B(0.000024968), COX7B(5.76E−05), ATP5G1(0.000552), ATP6V1B2(0.020631), UQCRFS1(4.82E−08), COX5A(9.06E−06), ATP12A(0.002001), ATP6V1B1(8.54E−11), COX5B(0.005213), ATP5G3(5.48E−05), NDUFB1(1.20E−06), ATP6V0B(3.18E−07), ATP6V0C(0.001556), NDUFS7(0.005646), NDUFS8(0.000199), ATP5L(6.10E−05), NDUFS3(0.001237), ATP6V0D1(0.000687), NDUFS2(9.52E−07), NDUFS1(0.000719), TCIRG1(2.68E−06), NDUFA4(6.21E−12), NDUFA3(0.000707), NDUFA4L2(0.000000), NDUFA6(3.74E−06), COX8A(2.45E−06), ATP6V1H(2.04E−09), NDUFA10(0.002332497), LHPP(1.05E−07), ATP6V1D(2.09E−05), PPA2(0.005748), SDHB(0.000273), ATP6V1A(0.000647), ATP6V0E2(1.46E−07), UQCRH(0.008671), NDUFV1(9.75E−06), SDHC(0.002922), SDHD(0.002733), ATP5C1(2.40E−06), ATP5A1(1.85E−07), ATP6V0A4(1.33E−15), UQCRB(0.000496)
map00250	Alanine, aspartate and glutamate metabolism	Metabolism; amino acid metabolism	Up	0.03679	12	GOT2(9.07E−07), GLUL(0.014290), GOT1(0.000478), ASS1(8.40E−11), ALDH5A1(9.42E−06), GLS(0.011416), ABAT(8.77E−11), ALDH4A1(3.26E−08), ASNS(0.005671), ASL(0.001059), AGXT(8.18E−05), DDO(0.002112)
map00260	Glycine, serine and threonine metabolism	Metabolism; amino acid metabolism	Up	4.36E−04	16	SHMT1(0.003744), SHMT2(4.22E−08), GATM(0.001104), AMT(3.71E−07), MAOA(0.000363), GCAT(2.42E−06), AGXT(8.18E−05), PIPOX(2.83E−09), GLDC(1.54E−08), ALAS1(4.18E−06), CTH(1.48E−06), DLD(0.001570), PHGDH(1.25E−05), DAO(5.49E−10), PSAT1(0.000768), SARDH(0.000146)
map00270	Cysteine and methionine metabolism	Metabolism; amino acid metabolism	Up	0.06916	12	GOT2(9.07E−07), ADI1(6.10E−05), LDHB(9.05E−10), CTH(1.48E−06), LDHA(5.38E−14), AHCY(6.74E−05), GOT1(0.000478), DNMT3L(0.000176), DNMT1(3.44E−06), AHCYL1(7.94E−05), AHCYL2(1.62E−08), MPST(0.000405)
map00280	Valine, leucine and isoleucine degradation	Metabolism; amino acid metabolism	Up	8.79E−14	33	HSD17B10(1.92E−07), ACADSB(1.06E−05), EHHADH(0.009979), ECHS1(1.41E−09), ACAT1(3.35E−05), ALDH3A2(0.001311), AUH(3.90E−05), MCCC2(0.01761787), MUT(2.71E−05), IVD(1.36E−05), OXCT1(1.70E−06), MCCC1(0.000919), ACAD8(1.38E−07), HADH(3.58E−10), HMGCL(2.12E−07), ALDH6A1(1.54E−09), ACAA2(0.015364), ACADM(0.000824944), ACADS(0.000351), BCKDHB(1.18E−06), DBT(0.000280), ALDH7A1(0.000678), HMGCS2(0.025830), ALDH1B1(1.21E−06), AOX1(0.000215), DLD(0.001570), ALDH2(2.04E−05), ABAT(8.77E−11), HIBCH(3.29E−05), PCCB(4.63E−09), PCCA(1.72E−07), ALDH9A1(7.68E−06), ACAA1(6.20E−09)
map00310	Lysine degradation	Metabolism; amino acid metabolism	Up	0.01017	17	DLST(1.58E−05), GCDH(2.55E−07), EHHADH(0.009979), OGDHL(7.95E−10), AASS(4.95E−05), ECHS1(1.41E−09), OGDH(0.015859), ACAT1(3.35E−05), ALDH3A2(0.001311), PIPOX(2.83E−09), ALDH7A1(0.000678), ALDH1B1(1.21E−06), PLOD2(7.01E−10), PLOD3(2.04E−06), ALDH2(2.04E−05), HADH(3.58E−10), ALDH9A1(7.68E−06)
map00330	Arginine and proline metabolism	Metabolism; amino acid metabolism	Up	1.16E−04	24	SAT1(0.003288), ALDH18A1(0.000317), ASS1(8.40E−11), GATM(0.001104), MAOA(0.000363), ASL(0.001059), ALDH3A2(0.001311), CKB(0.017515), GOT2(9.07E−07), ALDH7A1(0.000678), GLUL(0.014290), GOT1(0.000478), P4HA2(0.042440), P4HA1(5.46E−10), ALDH1B1(1.21E−06), CKMT2(6.95E−06), ARG2(1.53E−07), GLS(0.011416), ALDH2(2.04E−05), PRODH2(1.88E−10), ALDH4A1(3.26E−08), DAO(5.49E−10), OAT(1.18E−06), ALDH9A1(7.68E−06)
map00380	Tryptophan metabolism	Metabolism; amino acid metabolism	Up	0.00119	18	DDC(7.22E−06), GCDH(2.55E−07), KYNU(0.021680), MAOA(0.000363), EHHADH(0.009979), OGDHL(7.95E−10), ECHS1(1.41E−09), IDO1(5.75E−06), OGDH(0.015859), ACAT1(3.35E−05), ALDH3A2(0.001311), ALDH7A1(0.000678), ALDH1B1(1.21E−06), AOX1(0.000215), ALDH2(2.04E−05), CAT(0.000463), HADH(3.58E−10), ALDH9A1(7.68E−06)
map00410	beta-Alanine metabolism	Metabolism; metabolism of other amino acids	Up	0.00183	12	ALDH7A1(0.000678), ACADM(0.000825), ALDH1B1(1.21E−06), UPB1(0.030295), EHHADH(0.009979), ALDH2(2.04E−05), ABAT(8.77E−11), DPYS(3.24E−05), ECHS1(1.41E−09), HIBCH(3.29E−05), ALDH3A2(0.001311), ALDH9A1(7.68E−06)
map00620	Pyruvate metabolism	Metabolism; carbohydrate metabolism	Up	1.34E−06	23	LDHB(9.05E−10), LDHA(5.38E−14), ME2(0.009738), ACACB(0.015700), GRHPR(0.000187), PCK2(3.66E−08), ACAT1(3.35E−05), ALDH3A2(0.001311), PDHB(3.11E−07), PCK1(8.90E−06), HAGH(1.21E−06), ALDH7A1(0.000678), ALDH1B1(1.21E−06), PKM2(1.23E−05), PKLR(0.000109), AKR1B1(0.023956), DLD(0.001570), ALDH2(2.04E−05), PDHA1(3.64E−07), ALDH9A1(7.68E−06), MDH2(0.041430), MDH1(3.71E−05), PC(4.74E−08)
map00630	Glyo ylate and dicarbo ylate metabolism	Metabolism; carbohydrate metabolism	Up	0.01987	8	MTHFD1(0.007709), MTHFD2(0.001166), ACO2(0.009461), ACO1(3.37E−08), HAO2(1.30E−07), GRHPR(0.000187), MDH2(0.041430), MDH1(3.71E−05)
map00640	Propanoate metabolism	Metabolism; carbohydrate metabolism	Up	3.35E−08	22	ALDH6A1(1.54E−09), LDHB(9.05E−10), LDHA(5.38E−14), ACADM(0.000825), SUCLG2(0.001127), SUCLG1(7.68E−13), EHHADH(0.009979), ECHS1(1.41E−09), ACACB(0.015700), ACAT1(3.35E−05), ACSS3(0.016867), ALDH3A2(0.001311), ALDH7A1(0.000678), MUT(2.71E−05), ALDH1B1(1.21E−06), ALDH2(2.04E−05), ABAT(8.77E−11), HIBCH(3.29E−05), SUCLA2(0.002727), PCCB(4.63E−09), PCCA(1.72E−07), ALDH9A1(7.68E−06)
map00650	Butanoate metabolism	Metabolism; carbohydrate metabolism	Up	5.21E−06	20	ACADS(0.000351), ALDH5A1(9.42E−06), EHHADH(0.009979), ECHS1(1.41E−09), ACAT1(3.35E−05), ALDH3A2(0.001311), PDHB(3.11E−07), ACSM3(0.000166), ALDH7A1(0.000678), HMGCS2(0.025830), ALDH1B1(1.21E−06), OXCT1(1.70E−06), ALDH2(2.04E−05), ABAT(8.77E−11), BDH2(2.94E−05), PDHA1(3.64E−07), HADH(3.58E−10), BDH1(1.61E−05), ALDH9A1(7.68E−06), HMGCL(2.12E−07)
map00670	One carbon pool by folate	Metabolism; metabolism of cofactors and vitamins	Up	0.00782	9	MTHFD1(0.007709), TYMS(1.33E−06), MTHFS(5.33E−05), MTHFD2(0.001166), SHMT1(0.003744), SHMT2(4.22E−08), ALDH1L1(9.60E−05), AMT(3.71E−07), FTCD(9.75E−08)
map03320	PPAR signaling pathway	Organismal systems; endocrine system	Up	6.52E−04	27	ACOX2(9.21E−10), ACOX1(1.77E−07), CPT2(0.012060), EHHADH(0.009979), AQP7(7.03E−08), CYP4A22(5.91E−05), SORBS1(0.005358), APOC3(0.000893), ANGPTL4(6.66E−15), ACSL5(0.000549), ACADM(0.000825), SCD(2.00E−07), PCK2(3.66E−08), DBI(0.000967), CPT1A(0.028547), PCK1(8.90E−06), CYP4A11(3.26E−05), CD36(5.44E−06), HMGCS2(0.025830), FABP3(0.000589), FABP1(7.22E−12), GK(3.94E−07), FABP7(2.69E−05), SLC27A2(0.000151), FABP5(4.10E−10), FABP6(3.08E−06), ACAA1(6.20E−09)
map04510	Focal adhesion	Cellular processes; cell communication	Down	0.032781	53	PGF(0.000247), PDGFA(0.019680), TLN2(2.47E−07), PTEN(0.003678), PAK2(0.000209), PAK4(0.002613), SHC1(0.000611), PDGFD(0.000349), PRKCA(2.22E−05), EGFR(1.41E−07), ACTN1(0.000296), FLNA(0.035792), PRKCB(0.021118), VEGFB(0.020498), VEGFC(0.026817), MAPK1(2.74E−05), CCND1(5.27E−09), CRKL(0.007581), CCND3(0.000176), CCND2(0.000658), JUN(0.013527), VEGFA(1.13E−09), COL1A2(0.000143), PDGFRA(3.14E−06), LAMC1(1.19E−05), COL1A1(0.003965), CAV2(3.20E−12), CAV1(2.03E−12), ERBB2(3.60E−05), COL3A1(0.017713), ITGB1(4.97E−06), RAC2(1.03E−05), BCL2(0.040852), COL6A2(0.000499), PIK3R3(0.002499), LAMB1(0.004054), EGF(4.00E−10), FN1(3.26E−07), COL4A4(2.16E−08), COL4A2(0.000133), FLT1(3.53E−06), VAV3(0.002113), COL4A1(4.37E−07), MET(6.32E−06), MAPK10(2.71E−07), BIRC3(3.98E−07), COL5A2(0.000159), COL4A6(7.63E−11), KDR(0.001381), LAMA2(0.000494), VWF(9.83E−10), LAMA4(6.92E−08), ITGA5(9.13E−07)
map04512	ECM–receptor interaction	Environmental information processing; signaling molecules and interaction	Down	0.02571	26	COL3A1(0.017712), DAG1(0.000975), SDC4(0.006038), ITGB1(4.97E−06), SDC3(0.001060), CD47(0.000105), CD44(0.000548), COL6A2(0.000499), AGRN(0.040523), LAMB1(0.004054), FN1(3.26E−07), COL4A4(2.16E−08), COL4A2(0.000133), COL4A1(4.37E−07), HSPG2(0.001932), COL5A2(0.000159), COL4A6(7.63E−11), LAMA2(0.000494), VWF(9.83E−10), SDC1(0.040455), LAMA4(6.92E−08), CD36(5.44E−06), ITGA5(9.13E−07), COL1A2(0.000143), COL1A1(0.003965), LAMC1(1.19E−05)
map04514	Cell adhesion molecules (CAMs)	Environmental information processing; signaling molecules and interaction	Down	5.95E−04	44	HLA-DQB1(0.000000), CLDN16(1.72E−08), CLDN8(0.000000), MPZL1(0.000543), CLDN10(2.25E−05), ITGB2(7.34E−10), L1CAM(1.08E−05), HLA-DMB(1.83E−06), SDC4(0.006038), ITGB1(4.97E−06), CDH4(0.032744), HLA-DMA(1.04E−07), CDH5(0.000118), ITGAM(9.55E−07), SDC3(0.001060), VCAM1(0.001385), HLA-DRB4(0.000000), CD2(0.000423), CD4(0.000493), HLA-DPB1(3.92E−08), SELPLG(3.67E−06), SPN(0.039304), ICAM1(0.000321), PTPRC(1.12E−05), PTPRM(0.003162), ICAM2(0.021949), NLGN1(0.000208), NFASC(1.88E−06), CD99(2.14E−07), HLA-C(3.59E−06), HLA-B(8.79E−07), CD40(2.68E−07), HLA-E(3.29E−06), HLA-G(7.03E−06), HLA-F(5.75E−07), SIGLEC1(0.002946), SDC1(0.040455), CD86(1.91E−07), CD34(0.005288), PECAM1(6.83E−07), CD58(0.013779), HLA-DPA1(3.32E−07), VCAN(7.27E−05), JAM3(0.039287), HLA-DRA(8.95E−06)
map04610	Complement and coagulation cascades	Organismal systems; immune system	Up	0.00363	25	C7(3.66E−07), C3AR1(6.36E−08), A2M(0.015678), C3(9.80E−07), C5(6.29E−10), C1S(0.032201), BDKRB2(0.002440), KLKB1(0.020648), SERPINA5(3.33E−16), SERPINE1(0.002613), CFI(0.024368), CFD(8.97E−06), KNG1(0.000000), PLAT(0.000362), F11(2.44E−09), CR2(4.58E−05), F8(0.006901), SERPING1(0.032058), PLG(1.90E−09), PROC(9.00E−07), C1QA(4.11E−08), VWF(9.83E−10), C1QB(1.61E−09), PROS1(0.024909), F2R(0.034193)
map04612	Antigen processing and presentation	Organismal systems; immune system	Down	0.00605	28	HLA-DQB1(0.000000), PDIA3(0.039365), LGMN(0.002506), IFI30(0.006301), HLA-DMB(1.83E−06), HLA-DMA(1.04E−07), CANX(0.000217), CD74(0.000111), B2M(0.008700), TAPBP(6.91E−08), HSPA2(3.45E−06), TAP2(0.002398), TAP1(2.37E−08), HLA-DRB4(0.000000), HSPA6(1.12E−05), HSPA4(0.000390), CD4(0.000493), HLA-DPB1(3.92E−08), HSP90AA1(0.00004952), CREB1(0.039261), HLA-C(3.59E−06), CTSS(2.98E−06), HLA-B(8.79E−07), HLA-E(3.29E−06), HLA-G(7.03E−06), HLA-F(5.75E−07), HLA-DPA1(3.32E−07), CTSB(0.015631), HLA-DRA(8.95E−06)
map04666	Fc gamma R-mediated phagocytosis	Organismal systems; immune system	Down	0.03719	28	WASF3(0.000154), MARCKSL1(0.009354), WASF2(1.25E−05), ASAP2(0.017588), PIP5K1B(6.36E−05), ASAP1(1.36E−06), ASAP3(0.000381), ARPC5(2.53E−06), DOCK2(2.13E−06), RAC2(1.03E−05), GSN(0.000397), INPP5D(1.39E−05), PPAP2A(0.005090), PIK3R3(0.002499), PRKCA(2.22E−05), PTPRC(1.12E−05), VAV3(0.002113), SPHK2(4.65E−09), LYN(3.36E−05), HCK(4.00E−08), PRKCB(0.021118), MAPK1(2.74E−05), ARPC1B(1.77E−05), CRKL(0.007581), FCGR2B(0.007829), FCGR2C(5.76E−05), PLCG2(6.23E−08), MARCKS(4.86E−07), FCGR2A(2.11E−07)
map04940	Type I diabetes mellitus	Human diseases; endocrine and metabolic diseases	Down	0.01511	16	HLA-DQB1(0.000000), PRF1(9.91E−05), HLA-C(3.59E−06), HLA-B(8.79E−07), HLA-DMB(1.83E−06), HLA-E(3.29E−06), HLA-DMA(1.04E−07), HLA-G(7.03E−06), HLA-F(5.75E−07), CD86(1.91E−07), CPE(0.000140), HLA-DRB4(0.000000), HLA-DPA1(3.32E−07), HLA-DPB1(3.92E−08), HSPD1(0.002241), FAS(0.000865), HLA-DRA(8.95E−06)
map05010	Alzheimer’s disease	Human diseases; neurodegenerative diseases	Up	0.01525	46	HSD17B10(1.92E−07), UQCRC1(1.72E−10), NDUFB8(0.026633), ATP5B(0.000025), COX7B(5.76E−05), SNCA(8.65E−05), MME(5.58E−07), ATP5G1(0.000552), UQCRFS1(4.82E−08), COX5A(9.06E−06), COX5B(0.005213), ATP5G3(5.48E−05), NDUFB1(1.20E−06), NDUFS7(0.005646), TNFRSF1A(0.018183), CASP3(0.001836), APOE(0.002175), CALML3(6.10E−05), NDUFS8(0.000199), FAS(0.000865), CHP(1.02E−05), NDUFS3(0.001237), PLCB1(0.000313), GAPDH(1.55E−06), NDUFS2(9.52E−07), NDUFS1(0.000719), NDUFA4(6.21E−12), NDUFA3(0.000707), ADAM10(0.001821), NDUFA4L2(0.000000), NDUFA6(3.74E−06), COX8A(2.45E−06), CYCS(0.000302), NDUFA10(0.002332), ITPR3(0.001233), ITPR2(4.78E−09), MAPK1(2.74E−05), SDHB(0.000273), ATP2A2(0.003118), UQCRH(0.008671), NDUFV1(9.75E−06), SDHC(0.002922), SDHD(0.002733), ATP5C1(2.40E−06), ATP5A1(1.85E−07), UQCRB(0.000496)
map05012	Parkinson’s disease	Human diseases; neurodegenerative diseases	Up	5.80E−04	43	UQCRC1(1.72E−10), NDUFB8(0.026633), ATP5B(0.000025), SLC6A3(3.26E−05), SNCA(8.65E−05), UCHL1(0.000664), COX7B(5.76E−05), PINK1(0.009949), ATP5G1(0.000552), UQCRFS1(4.82E−08), COX5A(9.06E−06), COX5B(0.005213), ATP5G3(5.48E−05), NDUFB1(1.20E−06), NDUFS7(0.005646), CASP3(0.001836), HTRA2(0.016642), NDUFS8(0.000199), NDUFS3(0.001237), NDUFS2(9.52E−07), NDUFS1(0.000719), NDUFA4(6.21E−12), NDUFA3(0.000707), SLC25A4(0.001148), SLC25A5(5.04E−09), NDUFA4L2(0.000000), NDUFA6(3.74E−06), COX8A(2.45E−06), CYCS(0.000302), UBE2J1(0.002195), UBE2L6(1.49E−07), NDUFA10(0.002332), SDHB(0.000273), UBA1(0.018511), UQCRH(0.008671), SDHC(0.002922), NDUFV1(9.75E−06), PPID(0.016505), SDHD(0.002733), ATP5C1(2.40E−06), ATP5A1(1.85E−07), UBB(1.00E−04), UQCRB(0.000496)
map05110	Vibrio cholerae infection	Human diseases; infectious diseases	Up	0.01223	20	PRKCA(2.22E−05), TCIRG1(2.68E−06), ADCY3(2.20E−06), KDELR2(0.002836), ATP6V1H(2.04E−09), PDIA4(0.013818), ATP6V1B2(0.020631), ATP6V1B1(8.54E−11), ATP6V1D(2.09E−05), ATP6V0B(3.18E−07), PRKCB(0.021118), ATP6V0C(0.001556), ATP6V1A(0.000647), ATP6V0E2(1.46E−07), PLCG2(6.23E−08), ERO1L(5.50E−06), ATP6V0D1(0.000687), ATP6V0A4(1.33E−15), KCNQ1(2.22E−06), SEC61G(3.22E−11)
map05120	Epithelial cell signaling in Helicobacter pylori infection	Human diseases; infectious diseases	Up	0.02621	22	EGFR(1.41E−07), TCIRG1(2.68E−06), ADAM10(0.001821), LYN(3.36E−05), MET(6.32E−06), ATP6V1H(2.04E−09), ATP6V1B2(0.020631), MAPK10(2.71E−07), ATP6V1B1(8.54E−11), CCL5(0.000176), ATP6V1D(2.09E−05), ATP6V0B(3.18E−07), ATP6V0C(0.001556), ATP6V1A(0.000647), CASP3(0.001836), ATP6V0E2(1.46E−07), JUN(0.013527), PLCG2(6.23E−08), ATP6V0A4(1.33E−15), ATP6V0D1(0.000687), JAM3(0.039287), CHUK(0.001478)
map05212	Pancreatic cancer	Human diseases; cancers	Down	0.00669	25	PGF(0.000247), ERBB2(3.60E−05), TGFB1(1.25E−05), ACVR1B(0.020268), CDKN2A(6.57E−07), RAC2(1.03E−05), RALB(0.003717), TGFA(4.14E−06), EGF(4.00E−10), PIK3R3(0.002499), CHUK(0.001478), EGFR(1.41E−07), RALBP1(0.017196), ARHGEF6(1.56E−07), TP53(0.015394), MAPK10(2.71E−07), RB1(1.23E−05), STAT1(0.008747), RALGDS(0.001720), VEGFB(0.020498), VEGFC(0.026817), MAPK1(2.74E−05), CCND1(5.27E−09), VEGFA(1.13E−09), JAK1(0.003297)
map05219	Bladder cancer	Human diseases; cancers	Down	0.00612	17	EGFR(1.41E−07), FGFR3(0.004060), PGF(0.000247), ERBB2(3.60E−05), MMP9(0.013659), TP53(0.015394), RB1(1.23E−05), VEGFB(0.020498), VEGFC(0.026817), MAPK1(2.74E−05), TYMP(9.37E−07), CCND1(5.27E−09), CDKN1A(0.004616), CDKN2A(6.57E−07), VEGFA(1.13E−09), EGF(4.00E−10), MYC(9.64E−06)
map05222	Small cell lung cancer	Human diseases; cancers	Down	0.02571	26	CKS1B(5.60E−06), FHIT(3.52E−07), PTEN(0.003678), ITGB1(4.97E−06), BCL2(0.040852), PIK3R3(0.002499), LAMB1(0.004054), MYC(9.64E−06), TRAF5(0.010154), CHUK(0.001478), FN1(3.26E−07), COL4A4(2.16E−08), COL4A2(0.000133), COL4A1(4.37E−07), CYCS(0.000302), TP53(0.015394), SKP2(0.001476), RB1(0.041380), BIRC3(3.98E−07), COL4A6(7.63E−11), LAMA2(0.000494), LAMA4(6.92E−08), CCND1(5.27E−09), CDKN1B(8.75E−05), PIAS1(0.019851), LAMC1(1.19E−05)
map05320	Autoimmune thyroid disease	Human diseases; immune diseases	Down	0.08014	16	HLA-DQB1(0.000000), TG(0.001762), PRF1(9.91E−05), HLA-C(3.59E−06), CD40(2.68E−07), HLA-B(8.79E−07), HLA-DMB(1.83E−06), HLA-E(3.29E−06), HLA-DMA(1.04E−07), HLA-G(7.03E−06), HLA-F(5.75E−07), CD86(1.91E−07), HLA-DRB4(0.000000), HLA-DPA1(3.32E−07), HLA-DPB1(3.92E−08), FAS(0.000865), HLA-DRA(8.95E−06)
map05322	Systemic lupus erythematosus	Human diseases; immune diseases	Down	0.09307	27	HLA-DQB1(0.000000), C7(3.80E−07), C3(9.80E−07), C5(6.29E−10), SNRPD1(0.000590), C1S(0.032201), HLA-DMB(1.83E−06), HLA-DMA(1.04E−07), HIST1H2BK(1.05E−05), HLA-DRB4(0.000000), H2AFZ(0.001992), H2AFY(0.011433), HIST1H4C(0.025179), HLA-DPB1(3.92E−08), FCGR3B(8.53E−06), HIST1H2BE(0.009017), ACTN1(0.000296), CD40(2.68E−07), TRIM21(0.000120), C1QA(4.11E−08), C1QB(1.61E−09), CD86(1.91E−07), FCGR2B(0.007829), FCGR2C(5.76E−05), H2AFY2(0.000330), HLA-DPA1(3.32E−07), FCGR2A(2.11E−07), HLA-DRA(8.95E−06)
map05330	Allograft rejection	Human diseases; immune diseases	Down	0.00818	15	HLA-DQB1(0.000000), PRF1(9.91E−05), HLA-C(3.59E−06), CD40(2.68E−07), HLA-B(8.79E−07), HLA-DMB(1.83E−06), HLA-E(3.29E−06), HLA-DMA(1.04E−07), HLA-G(7.03E−06), HLA-F(5.75E−07), CD86(1.91E−07), HLA-DRB4(0.000000), HLA-DPA1(3.32E−07), HLA-DPB1(3.92E−08), FAS(0.000865), HLA-DRA(8.95E−06)
map05332	Graft-versus-host disease	Human diseases; immune diseases	Down	0.04031	14	HLA-DQB1(0.000000), PRF1(9.91E−05), HLA-C(3.59E−06), HLA-B(8.79E−07), HLA-DMB(1.83E−06), HLA-E(3.29E−06), HLA-DMA(1.04E−07), HLA-G(7.03E−06), HLA-F(5.75E−07), CD86(1.91E−07), HLA-DRB4(0.000000), HLA-DPA1(3.32E−07), FAS(0.000865), HLA-DPB1(3.92E−08), HLA-DRA(8.95E−06)
map05416	Viral myocarditis	Human diseases; cardiovascular diseases	Down	0.00107	27	HLA-DQB1(0.000000), PRF1(9.91E−05), CAV1(2.03E−12), DAG1(0.000975), ITGB2(7.34E−10), HLA-DMB(1.83E−06), HLA-DMA(1.04E−07), CASP3(0.001836), RAC2(1.03E−05), HLA-DRB4(0.000000), HLA-DPB1(3.92E−08), ICAM1(0.000321), CYCS(0.000302), HLA-C(3.59E−06), CD40(2.68E−07), HLA-B(8.79E−07), MYH9(0.011717), HLA-E(3.29E−06), MYH8(0.014687), HLA-G(7.03E−06), HLA-F(5.75E−07), LAMA2(0.000494), CD86(1.91E−07), CCND1(5.27E−09), HLA-DPA1(3.32E−07), MYH14(0.000268), HLA-DRA(8.95E−06), MYH10(0.002797)

## Discussion

Clear cell renal cell carcinoma is one of the most common types of kidney cancer in adult; however, its causes are not completely understood. The selection of differentially expressed genes and consistent pathways helps us to explore their underlying molecular mechanisms, thereby providing insights into biological function. Single gene-marker-based approaches can fail to detect transcriptional programs that are distributed across an entire network of genes are yet subtle at the level of individual gene [15]. Genome-wide microarrays can locate gene families and pathways which show a consistent alteration in a disease state. Pathway analysis is a valid method to reduce a major deviation and can obtain interesting common genes and pathways by mixing differently expressed genes from different datasets.

Some studies have been published. The study of Tun et al. [16] used gene expression profiling of early-stage ccRCC combined with a comprehensive bioinformatics analyses to reveal the significant pathway and transcription factors which take effect in the development of ccRCC. Meanwhile, Maruschke et al. [17] used microarray expression analysis to determine 16 gene sets that distinguish expression profiles from grade 1 and grade 3 tumor tissues based on MSigDB data bank analysis. The two studies above were single dataset analysis. However, multi-microarray dataset analysis for the development of ccRCC was rare. This study uses three datasets based on a novel GSEA carried out by KEGG dataset and meta-analysis approach to identify the common significant genes and genetic pathways with *p* < 0.05 associated ccRCC. And our findings suggest that most genes and pathways involved in ccRCC are the same according to their functional classification. In this study, we discussed several differentially expressed pathways and genes among crossing pathways which suggest the role of these pathways and genes in ccRCC based on their functional classification.

### Metabolism pathways

The metabolism pathways in our study were predominantly focused on carbohydrate metabolism, lipid metabolism, energy metabolism, amino acid metabolism, metabolism of other amino acids, metabolism of cofactors and vitamins. ccRCC is increasingly being recognized as a metabolic disease. Numerous studies have shown a significant association between body mass index, obesity and the development of kidney cancer [18]. In a case–control study from Iowa, diets richest in animal and saturated fats, oleic acid and cholesterol were associated with statistically significant increases in RCC (1.9–2.6-fold, depending on the factor) [19]. These metabolic abnormalities provide protection for the tumor but also may provide a source of vulnerability and therapeutic opportunity. Citrate cycle (TCA cycle) discovered in this study belongs to Carbohydrate metabolism; it is part of a metabolic pathway coupled to mitochondrial oxidative phosphorylation that converts nutrients to energy in aerobic cells. Recently, heterozygous germline mutations in fumarate hydratase (FH) or succinate dehydrogenase (SDH) of the TCA cycle have been shown to predispose individuals to tumors [20]. SDHB/C/D is the key gene in the Citrate cycle and oxidative phosphorylation pathway in our article. SDH is one of the seven known kidney cancer genes involved in pathways that respond to metabolic stress and/or nutrient stimulation [21]. Targeting the fundamental metabolic abnormalities in kidney cancer provides a unique opportunity for the development of more effective forms of therapy for this disease. Recently, early-onset renal tumors have been found to develop in individuals with germ line SDHB mutations [22, 23]. In preclinical models, increased succinate has been shown to inhibit HIF prolyl hydroxylase and affect HIF stability [24]. HIF can strengthen the expression of vascular endothelial growth factor (VEGF), glucose transcript factor 1 (GLUT-1) and glycolytic enzyme in the downstream target genes; promote the generation of blood vessels and energy metabolism of cells; and possibly play an important role in the developing progress in the excessive expression of malignant tumor [25, 26].

### Cellular processes and cell communication

**Fig. 1:**
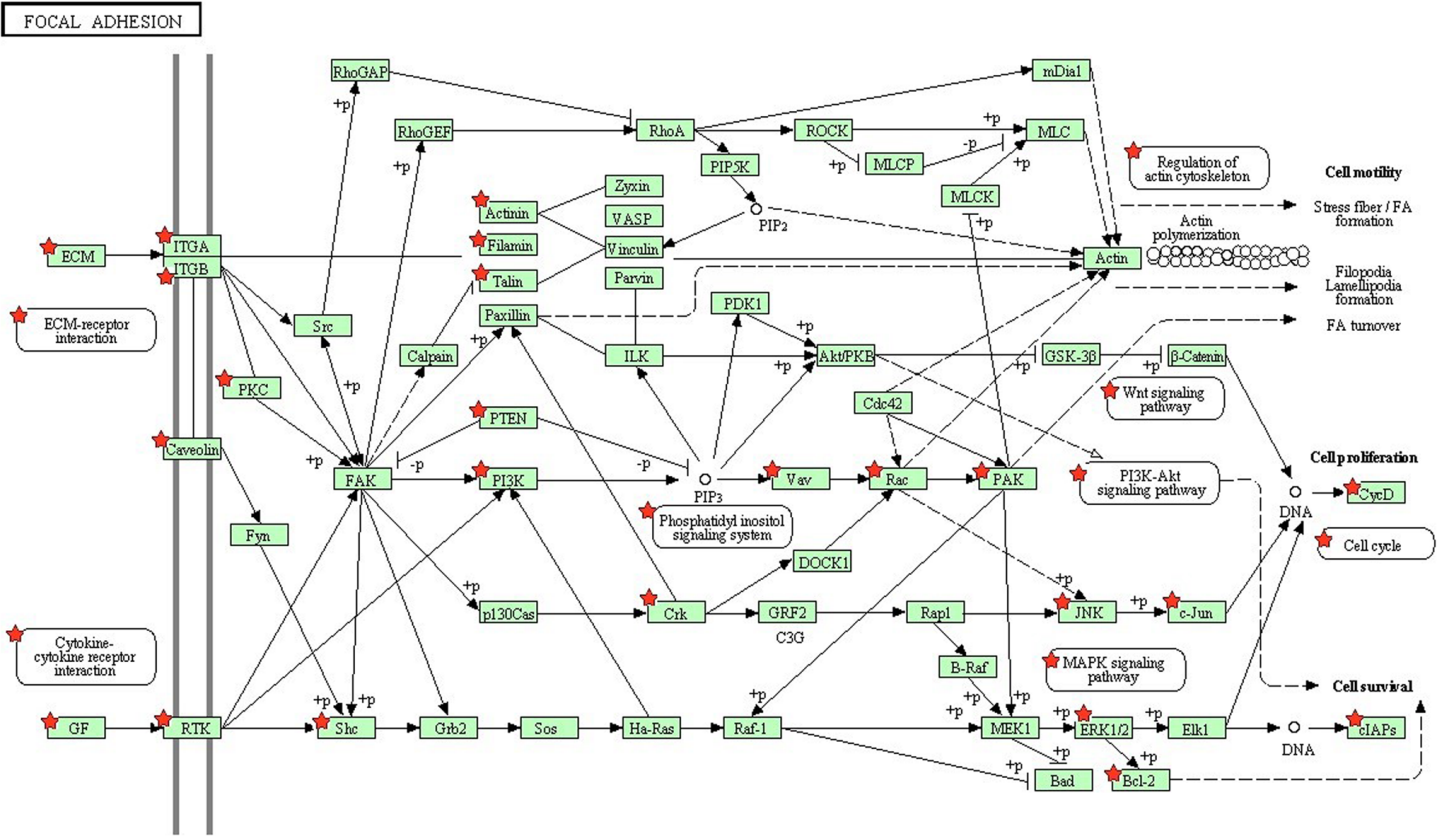
Focal adhesion pathway (the chart is from the KEGG database, ★*p* < 0.05, genes symbolized by ★ and correlation *p* values can be found in map04510 of Table 4)

Focal adhesion pathway (Fig. 1) in our result belongs to cellular processes and cell communication. In cell biology, focal adhesions (cell–matrix adhesions or FAs) are specific types of large macromolecular assemblies through which both mechanical force and regulatory signals are transmitted. Cell–matrix adhesions play essential roles in important biological processes including cell motility, cell proliferation, cell differentiation, regulation of gene expression and cell survival [27]. Tumor epithelial and endothelial cells require attachment to the extracellular matrix (ECM) for survival; on loss of adhesion, they undergo anoikis [28, 29]. Quinazoline-based drugs trigger anoikis in renal cancer cells by targeting the focal adhesion survival signaling. This potent antitumor action against human RCC suggests a novel quinazoline-based therapy targeting renal cancer [30]. VEGFA/B/C (marked by red stars in Fig. 1) is significant gene in focal adhesion pathway. The role of VEGF in particular has been explored as a key factor in the pathogenesis of RCC. VEGF functions to increase vascular permeability, induce endothelial cell proliferation and migration, and promote endothelial cell survival [31]. Furthermore, VEGF receptor expression has been observed in RCC cells, suggesting that VEGF may also serve as an autocrine stimulus in RCC [32]. The high VEGF expression in RCC is the direct result of inactivation of the Von Hippel–Lindau tumor suppressor gene (VHL). Data suggest that VHL inactivation occurs in the majority of ccRCC [33]. Therapeutic targeting of VEGF in RCC has strong biologic rationale. Substantial clinical activity has been reported in clinical trials with VEGF-targeting agents [34, 35]. Further investigation is needed to optimally use these agents for maximal clinical benefit.

### Environmental information processing

Extracellular matrix–receptor interaction and cell adhesion molecule (CAM) pathways were in this classification. They are all about signaling molecules and interaction. The extracellular matrix (ECM) consists of a complex mixture of structural and functional macromolecules and serves an important role in tissue and organ morphogenesis and in the maintenance of cell and tissue structure and function [36]. There is close connection between ECM–receptor interaction and focal adhesion pathway, which is also a significant pathway. At the cell–extracellular matrix contact points, specialized structures are formed and termed focal adhesions, where bundles of actin filaments are anchored to transmembrane receptors of the integrin family through a multi-molecular complex of junctional plaque proteins [37]. There is increasing evidence that certain integrins associate with receptor tyrosine kinases (RTKs) to activate signaling pathways that are necessary for tumor invasion and metastasis [38]. Zhou et al. [39] found that multiple canonical cancer-associated signaling pathways including focal adhesion, cell cycle and ECM–receptor interaction were significantly more likely to be disrupted in ccRCC than expected by chance. This is consistent with the results of our study.

CD47 is a key gene in ECM–receptor interaction pathway, which is involved in the increase in intracellular calcium concentration that occurs upon cell adhesion to extracellular matrix. As has been found by other investigators, CD47 over expression may be associated with ferric nitrilotriacetate-induced renal cortical tubular damage and regeneration that lead to a polycystic state, and with tumor progression and metastasis of the induced RCCs [40].

### Other pathways and genes

Human diseases and organismal systems are the two remaining classifications associated with ccRCC. Pathways such as type I diabetes mellitus, epithelial cell signaling in helicobacter pylori infection, bladder cancer, systemic lupus erythematosus and so on all belonged to the classification of human diseases. They are mainly about endocrine and metabolic diseases, neurodegenerative diseases, infectious diseases, cancers, immune diseases and cardiovascular diseases. Some diseases above belong to endocrine or immune system organic system in the classification of organismal systems. Most of genes in these pathways can be enriched in the above pathways. HLA-DQB1 appears in 8 pathways in human diseases, organismal systems and environmental information processing classification is an important gene for ccRCC, and this has widely been reported in the literature. Patients with RCC whose tumors did not express HLA-DQA1 or HLA-DQB1 molecules demonstrated poor clinical response [41]. EGFR and VEGFA/B/C expression in human disease pathways play an important regulatory role in tumor angiogenesis, invasion and metastasis. Based on the EGFR/VEGF target in the treatment of cancer is the hot spot in drug research [42].

## Conclusion

The pathogenesis of ccRCC is quite complicated. It is effective to identify differentially expressed genes and deduce their underlying molecular pathways based on gene set enrichment analysis and meta-analysis. The significant genes and pathways were mainly focused on metabolism, cellular processes and cell communication, environmental information processing, human diseases and organismal systems. They may have some connections with ccRCC. Furthermore, we verified some of the results by searching the literature in the discussion section. The conclusion is relatively reliable and can be used to guide further study. Further experiments are needed to verify specific links between these results and ccRCC.
